# A three-dimensional (3D), serum-free, Collagen Type I system for chondrogenesis of canine bone marrow-derived multipotent stromal cells (cMSCs)

**DOI:** 10.1371/journal.pone.0269571

**Published:** 2022-06-09

**Authors:** Melissa A. MacIver, Lauren K. Dobson, Carl A. Gregory, Ken Muneoka, W. Brian Saunders

**Affiliations:** 1 Department of Small Animal Clinical Sciences, College of Veterinary Medicine and Biomedical Sciences, Texas A&M University, College Station, Texas, United States of America; 2 Department of Molecular and Cellular Medicine, Texas A&M College of Medicine, Texas A&M University, College Station, Texas, United States of America; 3 Department of Veterinary Physiology and Pharmacology, College of Veterinary Medicine and Biomedical Sciences, Texas A&M University, College Station, Texas, United States of America; Friedrich-Loeffler-Institute, GERMANY

## Abstract

The dog is an underrepresented large animal translational model for orthopedic cell-based tissue engineering. While chondrogenic differentiation of canine multipotent stromal cells (cMSCs) has been reported using the classic micromass technique, cMSCs respond inconsistently to this method. The objectives of this study were to develop a three-dimensional (3D), serum-free, Collagen Type I system to facilitate cMSC chondrogenesis and, once established, to determine the effect of chondrogenic growth factors on cMSC chondrogenesis. Canine MSCs were polymerized in 100 μL Collagen Type I gels (5 mg/mL) at 1 x 10^6^ cells/construct. Constructs were assessed using morphometry, live/dead staining, and histology in 10 various chondrogenic media. Four media were selected for additional in-depth analyses via lactate dehydrogenase release, total glycosaminoglycan content, qPCR (*COL1A1*, *COL2A*, *SOX9*, *ACAN*, *BGLAP and SP7*), immunofluorescence, and TUNEL staining. In the presence of dexamethasone and transforming growth factor-β3 (TGF-β3), both bone morphogenic protein-2 (BMP-2) and basic fibroblast growth factor (bFGF) generated larger chondrogenic constructs, although BMP-2 was required to achieve histologic characteristics of chondrocytes. Chondrogenic medium containing dexamethasone, TGF-β3, BMP-2 and bFGF led to a significant decrease in lactate dehydrogenase release at day 3 and glycosaminoglycan content was significantly increased in these constructs at day 3, 10, and 21. Both osteogenic and chondrogenic transcripts were induced in response to dexamethasone, TGF-β3, BMP-2 and bFGF. Collagen Type II and X were detected in all groups via immunofluorescence. Finally, TUNEL staining was positive in constructs lacking BMP-2 or bFGF. In conclusion, the 3D, serum-free, Collagen Type-I assay described herein proved useful in assessing cMSC differentiation and will serve as a productive system to characterize cMSCs or to fabricate tissue engineering constructs for clinical use.

## Introduction

Since their initial discovery, mesenchymal stem cells, also referred to as multipotent stromal cells or MSCs, have received much interest as reparative agents for articular (hyaline) cartilage [1,2]. This interest stems from the ability of MSCs to undergo chondrogenic differentiation in both in vitro and in vivo assays [1,3,4], the prevalence of cartilage injury and osteoarthritis [5], and the inherently poor reparative capacity of articular cartilage [6–8]. Articular cartilage tissue engineering aims to repair replace, or restore normal structure and function through application of cells, scaffolds, and/or growth factors as implantable devices [1,9]. MSCs are a potential cell source for cartilage tissue engineering due to their presence in a variety of tissues, ease of harvest, and ability to differentiate into chondrocytes.

One limitation to articular cartilage tissue engineering is the inability to translate promising strategies from rodent or rabbit models to the clinical setting. Large animal translational models such as the goat, sheep, pig, and dog are used to bridge the gap between rodents and humans [10–12]. The dog is an excellent model species for orthopedic tissue engineering studies. Dogs are genetically diverse [13], experience similar biomechanical environments to humans [14,15], and can be assessed over time with non-invasive outcome measures that are used in human clinical trials [16,17].

In order to maximize the utility of the canine translational model in articular cartilage tissue engineering studies, chondrogenic differentiation of canine MSCs (cMSCs) must be more clearly defined. Although chondrogenesis of human MSCs (hMSC) is well-established [3,4,18], the effect of established differentiation methods on cMSC remains unclear [19,20]. Transforming growth factor-β (TGF-β) and bone morphogenic proteins (BMPs) are considered by many to be essential for chondrogenic differentiation of MSCs. A number of other growth factors have been assessed for chondrogenic effects [21]. Basic fibroblast growth factor (bFGF/FGF-2) is one of these growth factors [22,23]. In the context of cMSC chondrogenesis, Endo and colleagues recently described that bFGF improved chondrogenic differentiation of canine bone marrow peri-adipocyte cells (BM-PACs) [24]. This population of cells is somewhat unique. The effect of bFGF on chondrogenic differentiation of traditional cMSCs remains unknown.

Construct size is an additional issue preventing the translation of cartilage tissue engineering strategies to the clinical setting. Several techniques have been used to increase the size of tissue engineered cartilage. In a seminal study, Bhumiratana and colleagues fused multiple chondrogenic spheroids using a custom mold and press [25]. Synthetic and biologic matrices have also been used to fabricate larger chondrogenic constructs [1,17,26]. Of these, Collagen Type I has been assessed due to its biocompatibility, ease of isolation, and known ability to promote cell attachment and survival [1,3,27].

A long-term goal of our lab is to develop methods to enhance cMSC chondrogenesis and to subsequently generate clinically-sized chondrogenic constructs for tissue engineering in the canine translational model. The objectives of the present study were to first develop a three-dimensional (3D), serum-free, Collagen Type I in vitro system to facilitate cMSC chondrogenesis resulting in larger cMSC chondrogenic constructs and subsequently assess the effects of TGF-β, BMP-2, and bFGF on cMSC chondrogenesis.

## Materials and methods

### Isolation, characterization and culture of canine MSCs

Bone marrow-derived canine MSCs (cMSCs) were isolated from the iliac crest of an adult Walker Coonhound under animal use protocol (AUP 2011–149) and Institutional Animal Care and Use Committee supervision [28]. Using aseptic technique, 8 mLs of blood was aspirated under general anesthesia using a 12 gauge Illinois bone marrow biopsy needle into a 10 mL syringe pre-coated with 1 mL of 5000 IU/mL heparin. Heparinized marrow was immediately mixed with 3 mL of α-Minimum Essential Medium (α-ΜΕΜ, Invitrogen, Waltham, MA) in a sterile blood collection tube. Heparinized bone marrow sample was transferred to a 50 mL conical tube and diluted to 25 mLs with Hank’s Balanced Salt Solution (HBSS, Invitrogen). Next, 10 mLs of Ficoll-Paque (GE Health Care Biosciences, Piscataway, NJ) was placed into a new 50 mL conical tube and the 25 mL solution of bone marrow and HBSS were gently overlaid onto the Ficoll. Gradient centrifugation was performed at 1800xG for 30 minutes. The buffy coat was carefully isolated, diluted to 25 mLs with HBSS and centrifuged at 1000xG for 10 minutes. The supernatant was aspirated and the nucleated cells were resuspended in 2 mLs of PBS. Cells were plated at 1 x 10^7^ cells per 150 cm^2^ dish in 20 mLs of complete culture medium (CCM) consisting of α-MEM containing 100 units/mL penicillin, 100 μg/mL streptomycin, 0.292 mg/mL glutamate (Invitrogen) and 10% premium select fetal bovine serum (PS-FBS, Atlanta Biological, Inc., Flowery Branch, GA).

Cells were incubated overnight at 37°C and 5% humidified CO_2_. Plates were aspirated, washed with 10 mLs PBS, and re-supplied with CCM daily for three days. Media exchange was subsequently performed every other day until numerous primary colonies could be identified on each plate under phase contrast microscopy (approximately 60–70% confluence). The Passage 0 (P0) cells were washed in PBS, trypsinized, and re-plated at 250 cells/cm^2^ as Passage 1 (P1) cells. Upon reaching 70% confluence, P1 cells were confirmed to meet criteria for canine MSCs (cMSCs) as defined by Dominici et al. using assays optimized for cMSCs [28–30]. This included morphological assessment, colony forming unit (CFU) capacity, tri-lineage differentiation, cell surface profiling via flow cytometry, and RT-PCR for cell surface marker genes.

P1 cMSCs were cryopreserved in 30% PS-FBS (Atlanta Biological, Inc.), 5% tissue-culture grade DMSO (Sigma, St. Louis, MO), in α-MEM as 1 x 10^6^/mL aliquots and stored in liquid nitrogen. For experiments in the present study, P1 cMSCs (1 x 10^6^ total cells) were thawed and plated on two 150 cm^2^ tissue culture dishes in CCM and incubated overnight at 37°C and 5% humidified CO_2._ The following day cells were washed with PBS, trypsinized, and expanded as Passage 2 (P2) cells at 250 cells/cm^2^. Media changes were performed every other day until cells reached 70% confluence. Prior to each experiment, cMSCs were washed with PBS, trypsinized, and centrifuged at 500xG for 5 minutes. Cells were washed in Dulbecco’s Modified Eagle Medium (DMEM) (Invitrogen) to remove residual FBS from the medium in preparation for serum-free collagen type I 3D chondrogenic assays.

### Collagen Type I isolation

Collagen Type I was prepared from commercially acquired rat tail tendons using a non-proteolytic isolation method with sterile, filtered 0.1% acetic acid as previously described [31,32]. After lyophilisation, Collagen Type I was re-suspended at 7.1 mg/mL in 0.1% acetic acid and maintained at 4°C.

### Chondrogenic differentiation of cMSCs in 3D serum-free collagen constructs

**Fig 1:**
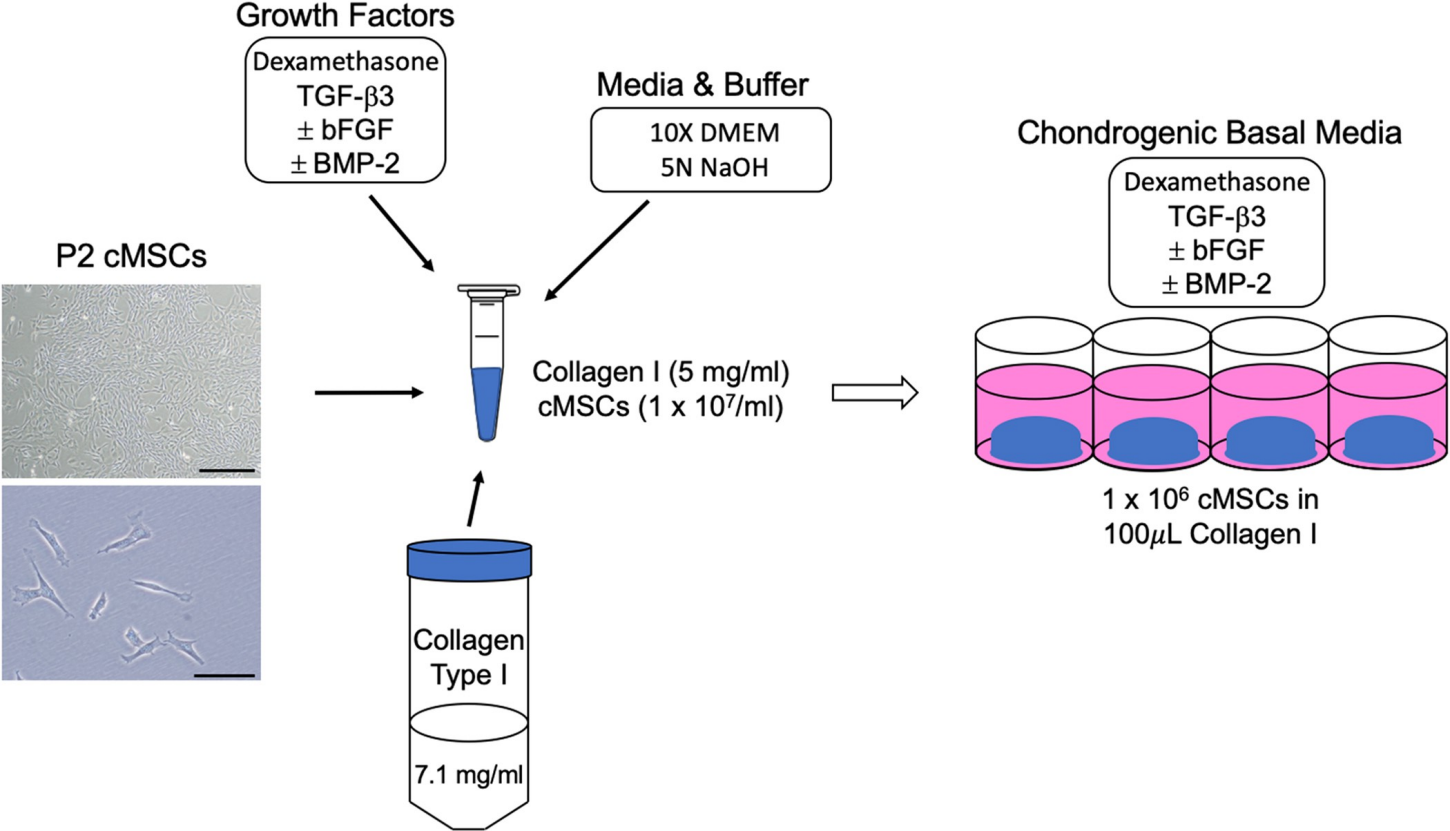
cMSC/Collagen Type I gel fabrication. Collagen Type I isolated from rat tail tendons (7.1 mg/mL) was pipetted into a new, chilled conical tube. Media and 5N NaOH (Table 1) were added at defined proportions and gently incorporated. Passage 2 cMSCs were trypsinized, washed in DMEM to remove FBS, and concentrated to 5 x 10^7^ cells/mL. cMSCs were added to the chilled collagen gel to a final concentration of 1 x 10^7^ cells/mL. Gels were supplemented with dexamethasone (1 x 10^−7^), 10 ng/mL TGF-β3 (rhTGF-β3, R&D Systems, Minneapolis, MN), and various combinations of rhBMP-2. Gels were pipetted in 100μL aliquots into 24-well plates, allowed to equilibrate at 37°C for 30 minutes, and supplied with chondrogenic basal medium containing growth factor combinations matching each collagen gel.

Collagen type I gels (100 μl volume) were prepared to a final collagen concentration of 5 mg/mL at 4°C as previously described [31]. Briefly, a stock gel was created for each experiment by pipetting 7.1 mg/mL Collagen Type I into a pre-chilled 50 mL conical tube. Next, 10x DMEM (Invitrogen), 5N NaOH, and additional 1x DMEM were added followed by gentle digital agitation. Canine MSCs and dexamethasone (Sigma) were added to the stock gel to achieve a final concentration of 1 x 10^7^ cMSCs/mL and 1 x 10^−7^ M dexamethasone. The stock gel was mixed gently via pipette and digital agitation taking care to avoid introduction of air bubbles. All reagents and cells were pre-chilled to 4°C and maintained on wet ice. Specific volumes of collagen, media, and cells were determined based on the total volume of gels needed for each set of experiments (Table 1). A schematic detailing the cMSC-collagen gel fabrication is provided in Fig 1.

**Table 1:** Three-dimensional (3D) Collagen Type I gel formula (5 mg/mL).

Component	0.5 mL gel	1 mL gel	2 mL gel	3 mL gel	4 mL gel	5 mL gel
**Collagen I**	352 μL	704 μL	1408 μL	2112 μL	2816 μL	3520 μL
**10X DMEM**	39.15 μL	78.3 μL	456.6 μL	234.9 μL	313.2 μL	391.5 μL
**5N NaOH**	2.1 μL	4.2 μL	8.4 μL	12.6 μL	16.8 μL	21 μL
**1X DMEM**	6.85 μL	13.7 μL	27.4 μL	41.1 μL	54.8 μL	68.5 μL
**cMSCs** **(5 x 10** ^**7**^ **/mL)**	100 μL	200 μL	400 μL	600 μL	800 μL	1000 μL

Table 1: Final volume of collagen gel and cell mixture was selected based on number of 100 μL cMSC/collagen constructs required per experiment (top row). Collagen Type I (4°C) was pipetted into a chilled 50 mL conical tube. Described amounts of 10X DMEM, 5N NaOH, and 1X DMEM (all 4°C**)** were incorporated. cMSCs (5 x 10^7^/mL) were added at a 1:5 dilution to achieve a final concentration of 1 x 10^7^ cells/mL.

Gels were supplemented with dexamethasone (1 x 10^−7^ M), 10 ng/mL TGF-β3 (rhTGF-β3, R&D Systems, Minneapolis, MN) and further aliquoted to include treatment groups containing various combinations of 50 or 500 ng/mL BMP-2 (rhBMP-2; R&D Systems) and 10 or 100 ng/mL of bFGF (rhbFGF, R&D Systems). Growth factor combination and concentrations were selected based on prior chondrogenesis studies [3,4,18,33]. After a final agitation, gels were pipetted in 100 μL volumes onto the center of 24 well plates such that each 3D chondrogenic construct contained 1 x 10^6^ cMSCs and Collagen Type I at a final concentration of 5 mg/mL. Plates were incubated at 37°C and 5% humidified CO_2_ for 30 minutes to initiate thermo-responsive gelation. Constructs were supplied with 750 μL/well of basal chondrogenic medium consisting of low-glucose DMEM with 100 units/mL of penicillin, 100 μg/mL of streptomycin, 1 x 10^−7^ M dexamethasone, a 1:250 dilution of reduced serum supplement-II (RSII), 50 μg/mL ascorbate-2-phosphate (Sigma), 40 μg/mL L-proline (Sigma), and 100 μg/mL sodium pyruvate (Sigma) [28,34,35]. Media were supplemented with TGF-β3, BMP-2, bFGF at concentrations matching each construct’s fabrication condition (Fig 1). All experiments were performed with a minimum of n = 3 constructs/condition, with up to n = 6 constructs/conditions based on individual assay needs. Cultures were incubated for up to 21 days with media exchange performed twice weekly.

### Experimental approach

**Fig 2:**
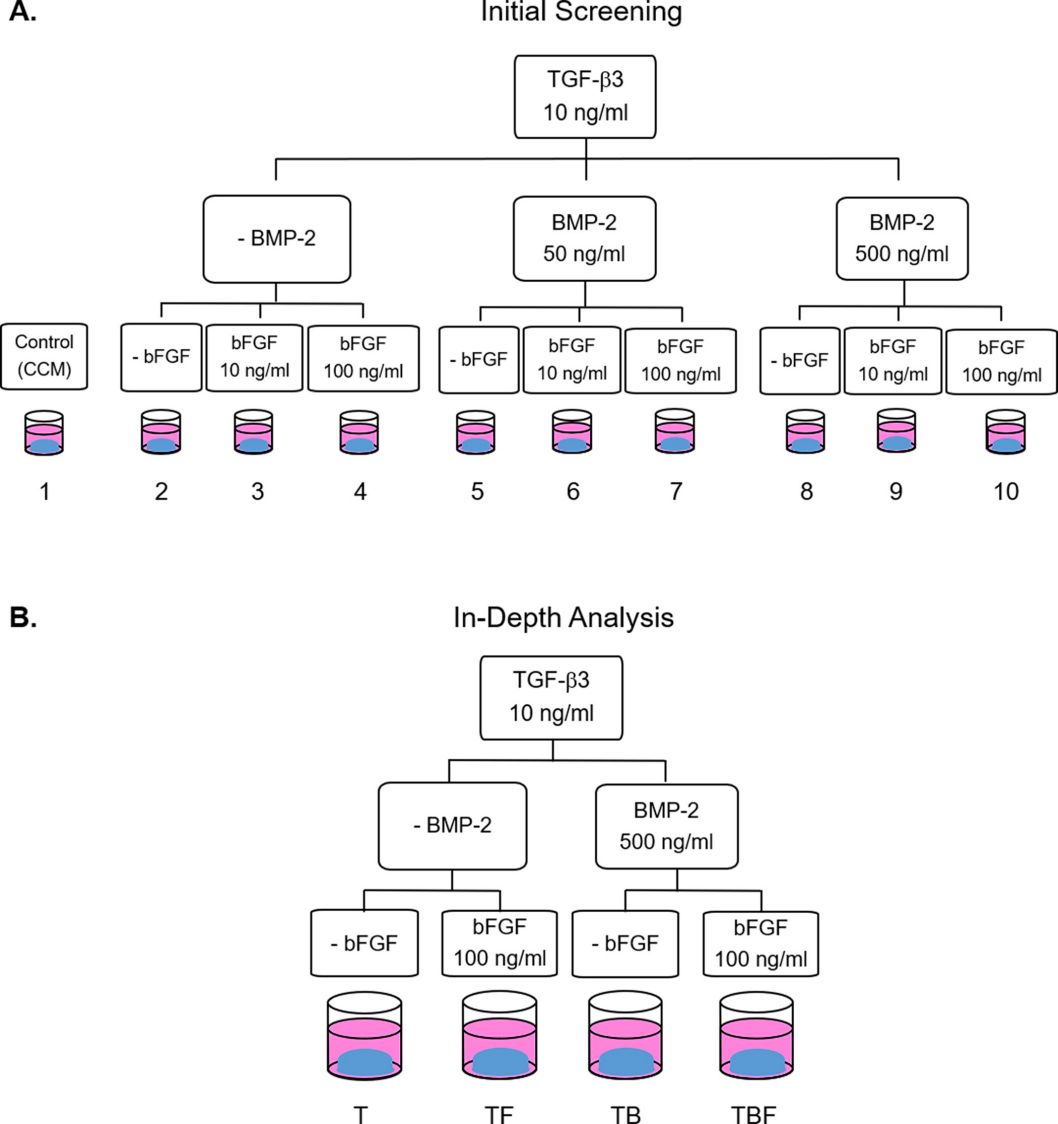
Experimental outline. A) In initial screening studies, 10 treatment conditions were assessed using gross photography, construct weight, and histology. For constructs cultured in chondrogenic media, chondrogenic basal media was supplemented with 10 ng/mL TGF-β3 and various combination of BMP-2 (50 or 500 ng/mL) and bFGF (10 or 100 ng/mL). Constructs were assessed by gross photography, construct weight, and 1% toluidine blue histology. B) Based on results from the screening studies described in panel A, four promising conditions were selected for in-depth analyses.

Numerous combinations and concentrations of growth factors have been reported to contribute to chondrogenesis in MSCs [21]. Based on historical impact on chondrogenesis, we elected to assess the effect of three growth factors on chondrogenesis of cMSCs in our 3D serum-free collagen system. Initial screening studies were performed with TGF-β3 (10 ng/mL) as well as BMP-2 (50 or 500 ng/mL) and bFGF (10 or 100 ng/mL). When combined with a CCM control, this resulted in 10 unique growth factor combinations. Fig 2A provides an overview of the treatment groups for the initial screening studies. Construct appearance, construct weight, and histology were used to identify promising cMSC chondrogenic conditions. For gross photography, individual constructs were washed with PBS, carefully removed from culture, and photographed at day 1, 3, and 21 to document change in construct shape and size. At day 3 and 21, residual moisture was removed from the surface of constructs with filter paper and construct weight was determined. At day 21, cultures were fixed in 10% neutral buffered formalin (NBF), embedded in paraffin, sectioned at 5μm, and stained with 1% toluidine blue for histology as previously described [34]. Informed by results of the initial screening studies, promising treatment groups were selected for in-depth analysis (Fig 2B).

### Subjective assessment of viability

At day 3, media were removed from constructs and two PBS washes performed. After the final wash, 500 μL of fresh PBS containing 0.2 μM calcein (Sigma) and 100 μg/mL propidium iodide (Botinium, Hawyard, CA) were added to each construct (n = 3 constructs/treatment condition). Constructs were incubated at 37°C for 30 minutes and evaluated for live/dead staining using an Olympus microscope and fluorescent microscopy. Individual fluorophore images were acquired. Subsequently, images were merged and overlays produced using SPOT software (version 5.1; Sterling Heights MI).

### Quantitative assessment of cytotoxicity

Chondrogenic constructs were cultured for either 1 or 3 days with media exchange occurring on day 1 and day 3 (after sample acquisition). Conditioned media (n = 3) from each treatment group were collected and stored at -20°C. Upon completion of all experiments, media were thawed on ice and assessed for LDH concentrations (a direct indicator of cytotoxicity) following manufacturer’s instructions (LDH Cytotoxicity Assay kit, ThermoScientific, Rockford, IL). LDH concentrations were determined by plotting values in conditioned media against a known standard curve.

### Quantitative assessment of sulfated proteoglycan and glycosaminoglycan (sGAG) content

Chondrogenic constructs were cultured for 3, 10 and 21 days to assess glycosaminoglycan content. At each time point, media were removed and n = 3 constructs per treatment condition were placed in 500 μL of 0.1 mg/mL papain extraction reagent based on manufacturer’s instructions (Blyscan^TM^ Assay, Biocolor Life Science Assays, County Antrim, UK). Constructs were heated at 65°C and manually agitated every 15–30 minutes until the constructs were no longer visible. Digests were then centrifuged at 10,000 RPM for 10 minutes and the supernatants were collected and stored at -20°C. Upon completion of the experiment, samples were allowed to thaw on ice and were assessed for sulfated proteoglycan and glycosaminoglycan content (sGAGs). Absorbance values were plotted against a standard curve of bovine tracheal chondroitin 4-sulfate to determine the concentration of sGAG within each chondrogenic construct.

### Quantitative real-time polymerase chain reaction (qPCR)

Constructs were cultured for 3, 10 and 21 days. RNA was isolated from cMSCs at day 0 prior to establishing 3D constructs in order to establish baseline transcription levels. At each time point, n = 6 cultures from each treatment group were washed in PBS and transferred to sterile 1.7 mL tubes containing 2.5 mg/mL bacterial collagenase (Sigma) in DMEM. Vented tubes were incubated at 37°C and 5% humidified CO_2_ for two hours. Constructs were manually agitated every 30 minutes until completely dissolved. Digests were centrifuged at 1,000 RPM for 10 minutes to pellet liberated cells. After removal of supernatants, messenger RNA (mRNA) samples were isolated using the Dynabeads ® mRNA Direct^TM^ Purification kit (ThermoFisher), treated with DNase to remove any contaminating DNA, and quantified using a Qubit Fluorometer 2.0 (ThermoFisher). Complimentary DNA (cDNA) was synthesized from 30 ng mRNA (normalized across all samples) using random hexamer primers and Superscript III reverse transcriptase (Invitrogen) following the SuperScript III RT kit instructions.

Canine qPCR primers for known osteogenic and chondrogenic genes were synthesized as follows: *COL1A1 (Collagen Type I)* [36] Forward: GCCGCTTCACCTACAGTGTCA, Reverse: GAGGTCTTGGTGGTTTTGTATTCG; *COL2A (Collagen Type II)* [36] Forward: CAGCAGGTTCACATATACTGTTCTGA, Reverse: CGATCATAGTCTTGCCCCACTT; *SOX9* [20] Forward: GCTCGCAGTACGACTACACTGAC, Reverse: GTTCATGTAGGTGAAGGTGGAG; *ACAN (Aggrecan)* [20] Forward: ATCAACAGTGCTTACCAAGACA, Reverse: ATAACCTCACAGCGATAGATCC; *SP7 (Osterix)* [20] Forward: ACGACACTGGGCAAAGCAG, Reverse: CATGTCCAGGGAGGTGTAGAC and *BGLAP (Osteocalcin)* [20] Forward: GAGGGCAGCGAGGTGGTGAG, Reverse TCAGCCAGCTCGTCACAGTTGG. Housekeeping genes *RPL13A* and *RPL32* were used and the genes were commercially synthesized as follows: *RPL13A* [37] Forward: GCCGAAGGTTGTAGTCGT, Reverse: GGAGGAAGGCCAGGTAATTC and *RPL32* [37] Forward: TGGTTACAGGAGCAACAAGAAA, Reverse: GCACATCAGCAGCACTTCA.

PCR reactions (20 μL) were prepared with 10 μL of SYBR Green Master Mix (ThermoFisher), 0.6 μL of Forward Primer, 0.6 μL of Reverse Primer, 6.8 μL of water, 0.4 μL of cDNA, and 1.6 μL of transfer RNA. Cycling conditions were performed using the CFX96 Real-Time System (Bio-Rad, Hercules, CA) with an initial SYBR® Green PCR Master Mix enzyme activation at 95°C for 10 minutes, followed by 40 cycles of: denature at 95°C for 15 seconds, anneal/extend at 60°C for 1 minute. A melt curve was performed immediately after qPCR. Cycling conditions were performed at 55°C for 30 seconds followed by 55°C for 5 seconds. Following this, the temperature was increased by 0.5°C per cycle per second for a total of eighty cycles.

cDNA was used in two independent qPCR reactions measuring the six genes of interest using *RPL13A* and *RPL32* as housekeeping genes. The expression for each gene of interest was averaged over the two independent qPCR reactions. The threshold (CT) levels were normalized to both the housekeeping genes and the gene expression at day 0 for relative gene expression using the 2^-ΔΔCt^ method [38]. Relative gene expression was plotted using GraphPad Prism 6.0 (GraphPad Software, La Jolla, CA).

### Histology

At 21 days of culture, constructs were removed from media, washed with 500 μL of PBS, and fixed in 500 μL of 10% neutral-buffered formalin for 24 hours. Constructs were then rinsed with PBS and dehydrated via increasing alcohol gradations, cleared with Sub-X clearing agent (Surgipath Medical, Richmond, IL) and embedded in paraffin (Richard-Allan Scientific, San Diego, CA). Paraffin embedded samples were cut to 4 μm sections, and floated onto Gold Seal Ultra stick slides (Thermo Scientific). Prior to staining, slides were heated to 60°C for 20 minutes in deparaffinized in HiPur Xylene (Thermo Scientific) and rehydrated through a series of alcohol gradients. Sections were stained with 1% toluidine blue (Ricca, Fort Worth, TX) or 0.1% Safranin O (IHC World, Woodstock, MD) with 0.05% Fast Green counterstain to assess proteoglycan deposition and cellular morphology.

#### Immunofluorescence

Immunofluorescence was performed for aggrecan, Collagen Type II, and Collagen Type X. Sections were warmed at 60°C for 20 minutes, deparaffinized in HiPur Xylene and rehydrated. Antigen retrieval was performed using 10 μL of proteinase K (Agilent) followed by a 15-minute incubation at 37°C [39]. Diluted normal goat serum was used for blocking for 60 minutes at room temperature in a PBS humidified chamber [Vectastain Elite ABC Kit (Rabbit IgG), Vector Laboratories, Burlingame, CA]. Primary antibodies were diluted in 1% BSA in sterile tris-buffered saline (TBS) at a 1:100 for Collagen II (PA-85108, Invitrogen), 1:200 for Collagen X (ab58632, Abcam) or 1:100 for aggrecan (138801AP; Proteintech Group, Inc, Rosemont, IL). Antibodies were applied in 20 μL volumes and incubated overnight at 4°C. Normal rabbit IgG was applied at the same concentrations as the primary antibodies to serve as negative controls. The following day, slides were washed with TBS including 0.1% Tween [39]. Visualization of fluorescent staining was assessed using goat anti-rabbit Alexaflour 488 (green) conjugated secondary antibody (Invitrogen) at 1:500 for one hour at room temperature. Nuclear fluorescence was visualized with a five-minute incubation of 4′,6-diamidino-2-phenylindole (DAPI, Invitrogen). Slides were rinsed in deionized water, dried, and mounted with Prolong Gold Antifade mountant (Invitrogen). Representative images were obtained using an Olympus IX70 fluorescence microscope using SPOT software (version 5.1; Sterling Heights, MO).

#### TUNEL staining

Paraffin-embedded sections were deparaffinized and rehydrated as described above. Sections were then analyzed for in situ cell death detection using the In Situ Cell Death Detection Kit, AP (Roche Applied Science, Germany). Following manufacturer’s instructions, positive control slides were created by applying recombinant DNase I for 10 minutes at room temperature to induce DNA strand breaks prior to the TUNEL labeling procedure. Negative control slides were incubated with 50 μL of label solution lacking terminal transferase. Positive control and treatment group slides were incubated with 50 μL of TUNEL reaction mixture containing Converter-AP and substrate solutions. Following TUNEL staining, slides were counterstained with 0.5% Light Green (Sigma).

### Statistical analysis

Descriptive statistics were reported as mean ± standard deviation for all continuous data sets. Analytical statistics included two-way ANOVA with Tukey’s post-hoc test. Descriptive and analytical statistics were performed with GraphPad Prism 6.0. Significance was established as p≤0.05.

## Results

### Characterization of canine MSCs

**Fig 3:**
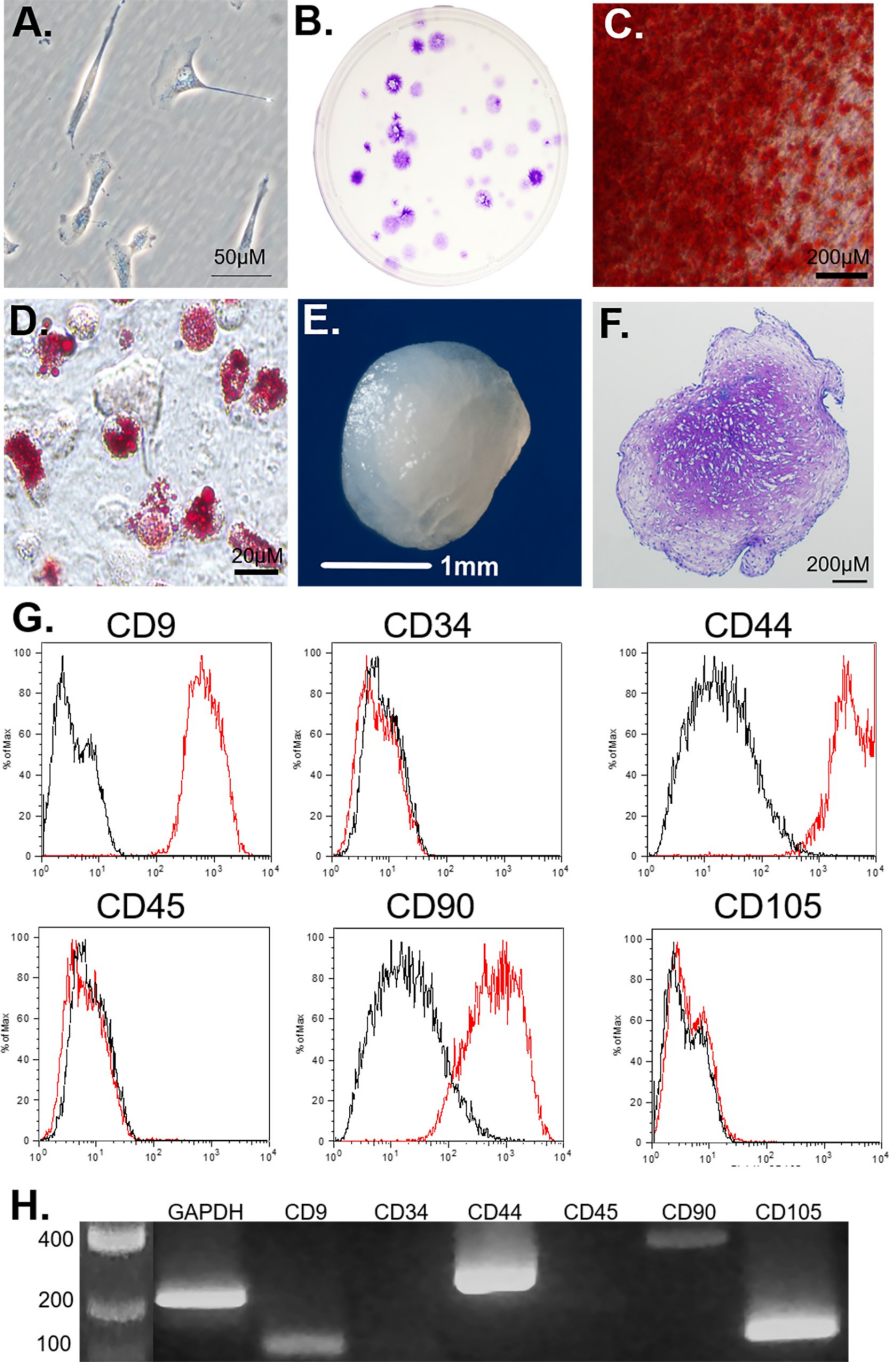
Characterization of passage 1 (P1) bone marrow-derived canine mesenchymal stromal cells (cMSCs). A) Cells exhibited the classic spindle-shaped morphology typical of MSCs. B) Cells were seeded at 100 cells per 150 cm^2^ tissue culture dish, incubated at 37°C without media exchange for 21 days, fixed and stained with toluidine blue to confirm colony forming unit (CFU) capacity. C) Cells were seeded at 1 x 10^4^ cells/cm^2^ and cultured in osteogenic differentiation medium containing 200 ng/mL rhBMP-2 with media exchange twice weekly. At 21 days, cells were fixed and stained with Alizarin Red stain to confirm calcium deposition within the monolayer. D) Cells were seeded at 1 x 10^4^ cells/cm^2^ and cultured in adipogenic medium for 21 days with media exchange twice weekly. Cells were fixed and stained with Oil Red O to document accumulation of intracellular vacuoles containing lipid. E,F) 2.5 x 10^5^ cells were centrifuged and cultured in chondrogenic induction medium as previously described for 21 days with media exchange twice weekly. Constructs were photographed (E), fixed, and sectioned for histology and toluidine blue staining (F) to confirm proteoglycan accumulation. G) Cells were assessed for cell-surface markers using flow cytometry. Cells were CD9^+^, CD34^-^, CD44^+^, CD45^-^, CD90^+^, and CD105^-^. H) RNA was isolated from cMSCs and assessed via RT-PCR for the cell surface markers evaluated in panel G. RT-PCR results were consistent with flow cytometry, with the exception of CD105^-^, which was present on RT-PCR and absent on flow cytometry.

The P1 canine bone marrow-derived cells were characterized as described above and met established criteria for MSCs (Fig 3) [28,29]. Cells were plastic adherent, exhibited a spindle-shaped morphology, and were capable of forming large colonies of cMSCs in CFU assays (Fig 3A and 3B). The cells were capable of tri-lineage differentiation (Fig 3C–3F) as assessed by osteogenic, adipogenic, and chondrogenic assays. When assessed by flow cytometry (Fig 3G), cells were CD9^+^, CD34^-^, CD44^+^, CD45^-^, CD90^+^, CD105^-^. RT-PCR for cell surface marker genes were consistent with flow cytometry results (Fig 3H), with exception of CD105 which was detected with RT-PCR.

### Screening experiments

#### Gross photography and construct weight

**Fig 4:**
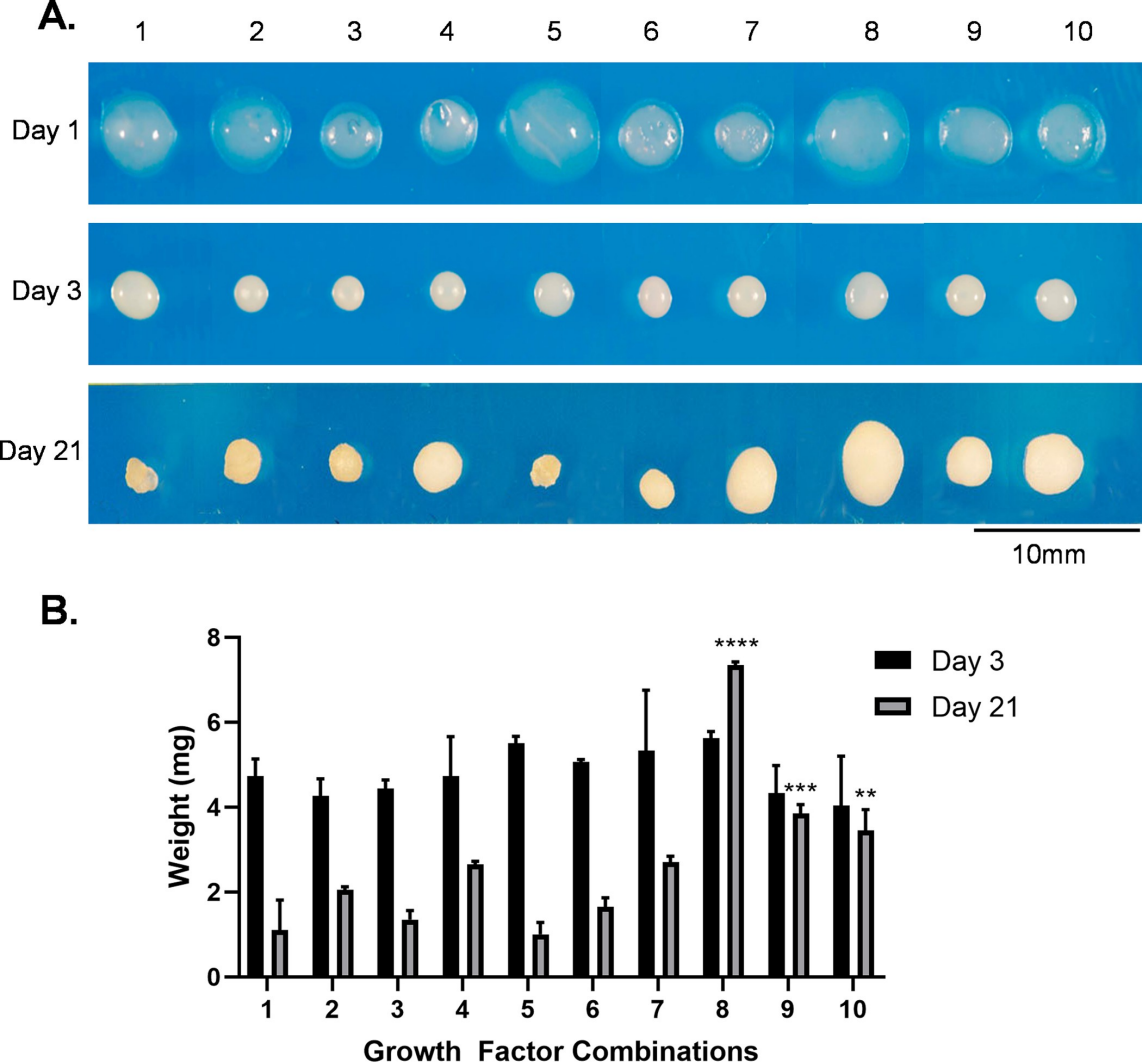
Gross photography and construct weight for initial chondrogenic screening. A) At day 1, 3, and 21, constructs were washed in PBS and transferred to a back-lit table for gross photography. B) At day 3 and 21, constructs (n = 3/condition) were dried to remove surface moisture and weighed to determine construct weight. No significant differences were detected across the treatment groups for day 3 construct weights. At day 21, treatment groups 8,9, and 10 were significantly greater than groups 1–7, represented by **** (p<0.001), *** (p = 0.008), and ** (p = 0.044). Within treatment groups, there were significant differences in construct weight between day 3 and day 21 for groups 1–8; however, there were no differences between day 3 and day 21 weights for groups 9 (p = 0.9933) and 10 (p = 0.9734).

Representative photographs of constructs treated with the conditions described in Fig 2A are shown in Fig 4A. At day 1, constructs remained somewhat similar in shape and size to their appearance immediately upon gel polymerization. Treatment groups lacking bFGF (1,2,5,8) were subjectively larger and groups containing bFGF and 0 or 50 ng/mL BMP-2 (3,4,6,7) were subjectively smaller. By day 3, all constructs underwent a dramatic condensation event.

By day 21, constructs cultured in CCM (1) or low concentrations of both BMP-2 and/or bFGF (groups 2,3,5,6) exhibited a smaller size and irregular shape. In contrast, cultures treated with higher concentrations of bFGF and/or BMP-2 (groups 4,7,8,9,10) were larger in size and more uniformly spherical in shape (Fig 4A). These observations suggested that chondrogenic basal medium containing TGF-β3 (10 ng/mL) and higher concentrations of BMP-2 and/or bFGF were beneficial for maintaining construct shape and size. Construct weight was assessed at day 3 and day 21 (Fig 4B). There was a consistent decrease in construct weight at day 21, with the exception of treatment group 8 (10 ng/mL TGF-β3, 500 ng/mL BMP-2, and 0 ng/mL bFGF). Construct weights for treatment groups 9 and 10 (10 ng/mL TGF-β3, 500 ng/mL BMP-2, and 10 or 100 ng/mL bFGF) remained consistent between day 3 and day 21. These results suggested that constructs cultured in 500 ng/mL BMP-2 were consistently the largest and heaviest at the day 21 and as such the 500 ng/mL BMP-2 concentration was selected for in depth analyses.

#### Histology

**Fig 5:**
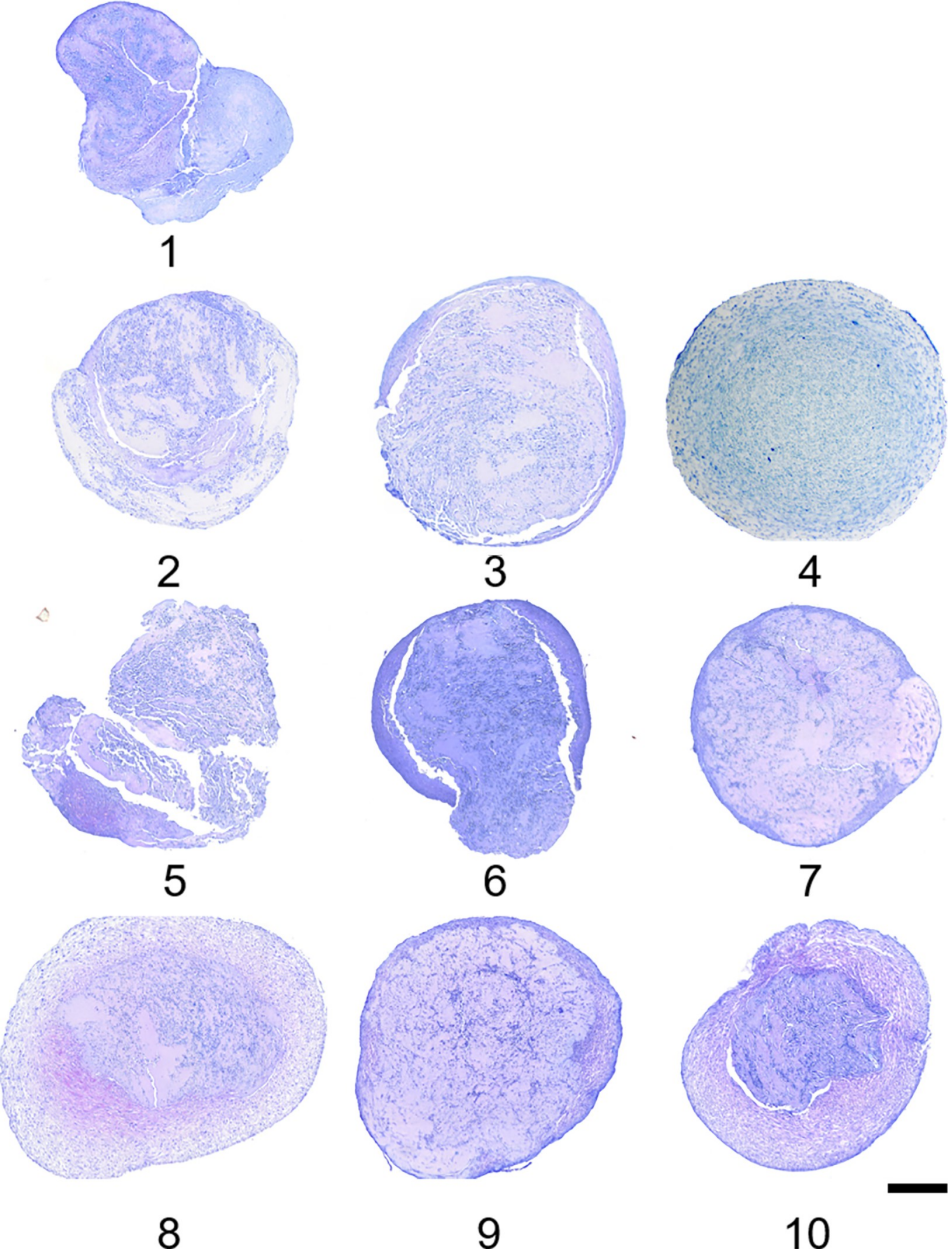
Toluidine blue histology of cMSC constructs for initial chondrogenic screening. Constructs from Fig 4 were fixed in 10% NBF, processed, embedded, cut at 4 μm sections, and stained in 1% toluidine blue. Representative images are provided for each of the 10 treatment groups. Groups containing the low concentrations of bFGF and/or BMP-2 exhibited irregular margins, large intra-construct defects, limited proteoglycan staining, and inconsistent distribution of cMSCs (groups 1,2,3,4,6). Treatment groups containing the high concentrations of bFGF or BMP-2 were larger, with smooth margins, enhanced proteoglycan deposition, and more uniformly distributed cMSCs (purple coloration, groups 4,8,10). Bar = 500 μm.

Representative toluidine blue histologic images are provided in Fig 5. Constructs cultured in CCM (group 1), without BMP-2 and bFGF (group 2), or with low concentrations of bFGF (group 3) were irregular in shape, with large defects present throughout the constructs. Cellular distribution for treatment groups 1–3 was irregular, and proteoglycan deposition was limited. Constructs cultured in low concentrations of BMP-2 (50 ng/mL) and 0 or 10 ng/mL of bFGF were similar in histologic appearance (groups 5,6). Group 7 constructs, which were cultured in 50 ng/mL BMP-2 and 100 ng/mL of bFGF, were spherical, lacked large intra-construct defects; however, proteoglycan deposition remained weak and cellular distribution was irregular. Treatment groups containing 10 ng/mL TGF-β3 with the combination of 100 ng/mL bFGF or 500 ng/mL BMP-2 contained the greatest number of cells and more uniform proteoglycan deposition (groups 4, 8, and 10).

Based on the results of initial screening experiments, treatment groups 2 (10 ng/mL TGF-β3), 4 (10 ng/mL TGF-β3, 100 ng/mL bFGF), 8 (10 ng/mL TGF-β3, 500 ng/mL BMP-2), and 10 (10 ng/mL TGF-β3, 500 ng/mL BMP-2, and 100 ng/mL bFGF) were selected for in-depth analyses. These groups contained the higher concentrations of bFGF and BMP-2 and for the in-depth analyses, were referred to as T, TF, TB, and TBF, respectively (Fig 2B). While treatment group 2 was not a strong performer in initial screening studies, it was selected to serve as the control condition for in-depth analyses based on classic chondrogenic differentiation literature in which chondrogenesis was initiated with medium containing TGF-β but lacking BMPs [40].

### In-depth analysis

#### Assessment of early viability with live/dead staining and lactate dehydrogenase (LDH) release

**Fig 6:**
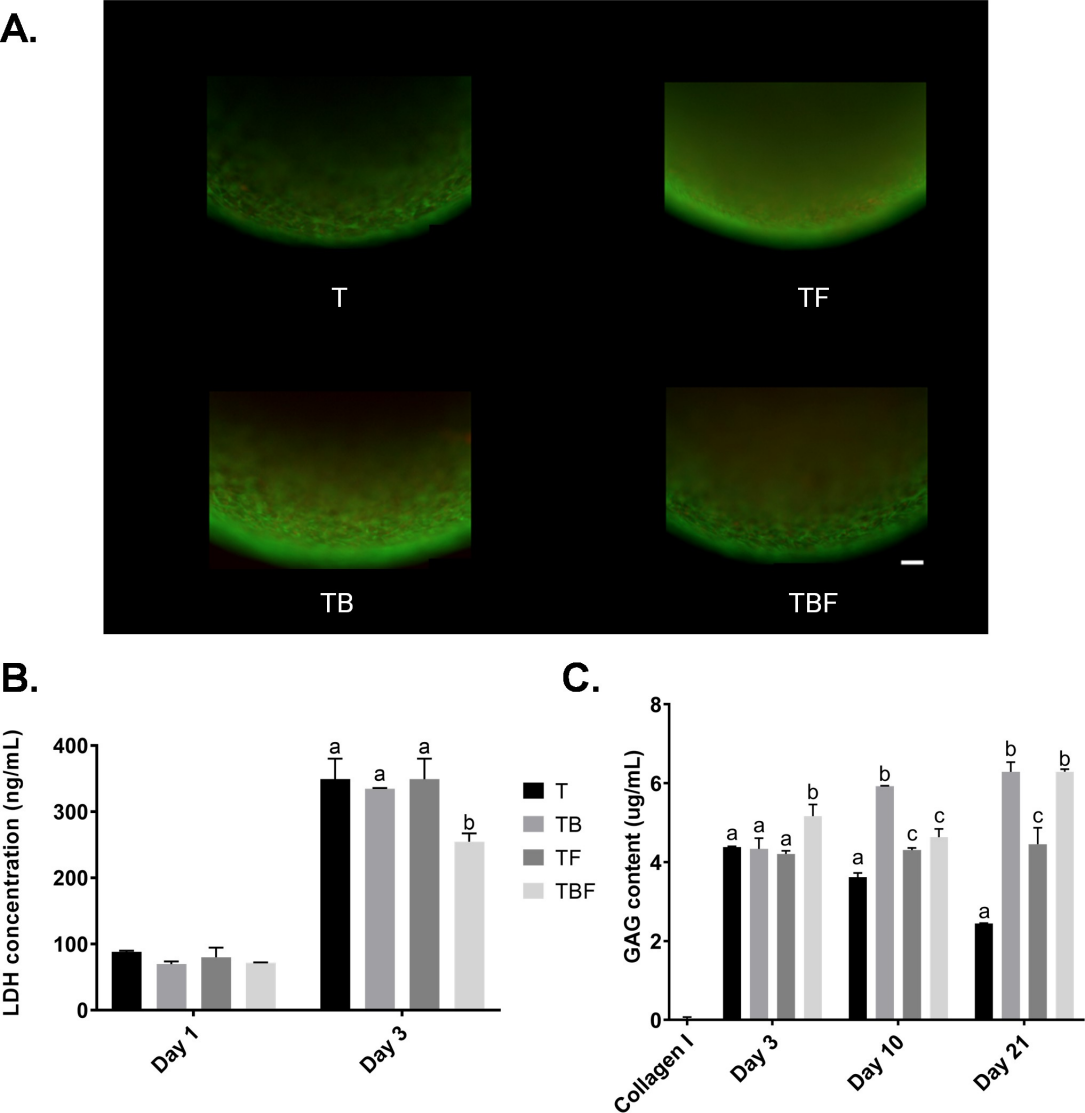
Cytotoxicity and total glycosaminoglycan (GAG) content. Constructs were fabricated as described in Fig 2B and cultured for up to 21 days. A) At day 3, constructs were assessed for cytotoxicity using live/dead staining with calcein (green, live) and propidium iodide (red, dead). While occasional dead cells were identified, the vast majority of cells were viable. Bar = 100 μm. B) Conditioned media were collected at day 1 and at day 3 to assess cytotoxicity using the lactate dehydrogenase (LDH) assay. At day 1 (24 hours conditioning), significant differences were not detected between treatment groups. At day 3 (48 hours conditioning), LDH concentrations were higher than day 1 in all groups. Treatment group TBF had a lower LDH concentration than other groups. C) Constructs were digested in papain solution prior to assessment of total GAG content at day 3, 10, and 21. Constructs cultured with BMP-2 and/or bFGF exhibited the highest total GAG concentrations.

In order to assess viability and cell stress during early chondrogenic culture, constructs were evaluated with live/dead staining at day 3. Representative 10X objective overlay images of live and dead fluorescence indicated that the majority of the imaged cells were viable as determined by strong calcein (green) signal (Fig 6A). In contrast, cells staining with propidium iodide (red) were only occasionally noted. Subjectively, there was minimal difference between the four treatment groups.

Cytotoxicity of the cMSC/collagen gel constructs was quantitatively assessed by assaying LDH concentrations in conditioned media at day 1 and day 3 (Fig 6B). There was a significant increase in LDH at day 3 as compared to day 1 (p<0.0001). When comparing treatment groups within each day, there were no significant differences in LDH concentrations on day 1. At day 3, LDH concentrations in treatment group TBF was significantly lower than group T (p = 0.0024), group TB (p = 0.0067), and group TF (p = 0.0024). Importantly, at day 1 media were conditioned for 24 hours whereas at day 3 media were conditioned for 48 hours. These results suggest that constructs treated with 10 ng/mL TGF-β3, 500 ng/mL BMP-2, and 100 ng/mL bFGF (group TBF) exhibited reduced cell stress during early stages of culture.

#### Quantitative assessment of total sulfated glycosaminoglycan (GAG) content

Deposition and accumulation of sulfated glycosaminoglycan and proteoglycan is a gold standard metric of chondrogenic differentiation. Total sulfated GAG content was quantified at day 3, day 10, and day 21 (Fig 6C). There was no detectible GAG in the Collagen Type I used to fabricate constructs (Collagen I). There was a significant increase in total GAG over the 21-day time course (p = 0.0096). At day 3, GAG concentration was significantly higher in treatment group TBF as compared to group T (p = 0.0083), TB (p = 0.0056), and TF (p = 0.0019). At day 10, GAG content was significantly greater in group DTB as compared to groups T (p<0.0001), TF (p<0.0001), or TBF (p = 0.0001). At day 10, group T exhibited lower GAG content as compared to TB (p<0.0001), TF (p = 0.0206), and TBF (p = 0.0012). At day 21, GAG concentration was significantly greater in groups containing BMP-2 (TB and TBF) as compared to group T (p<0.0001) and TF (p<0.0001). At 21 days, group T exhibited the lowest GAG concentration when compared to TB (p<0.0001), TF (p<0.0001), and TBF (p<0.0001). These results demonstrate that in this serum-free, 3D, Collagen Type I system, GAG content increased over time and that treatment groups TB and TBF exhibited the highest total GAG content over time.

#### Quantitative assessment of gene expression with qPCR

**Fig 7:**
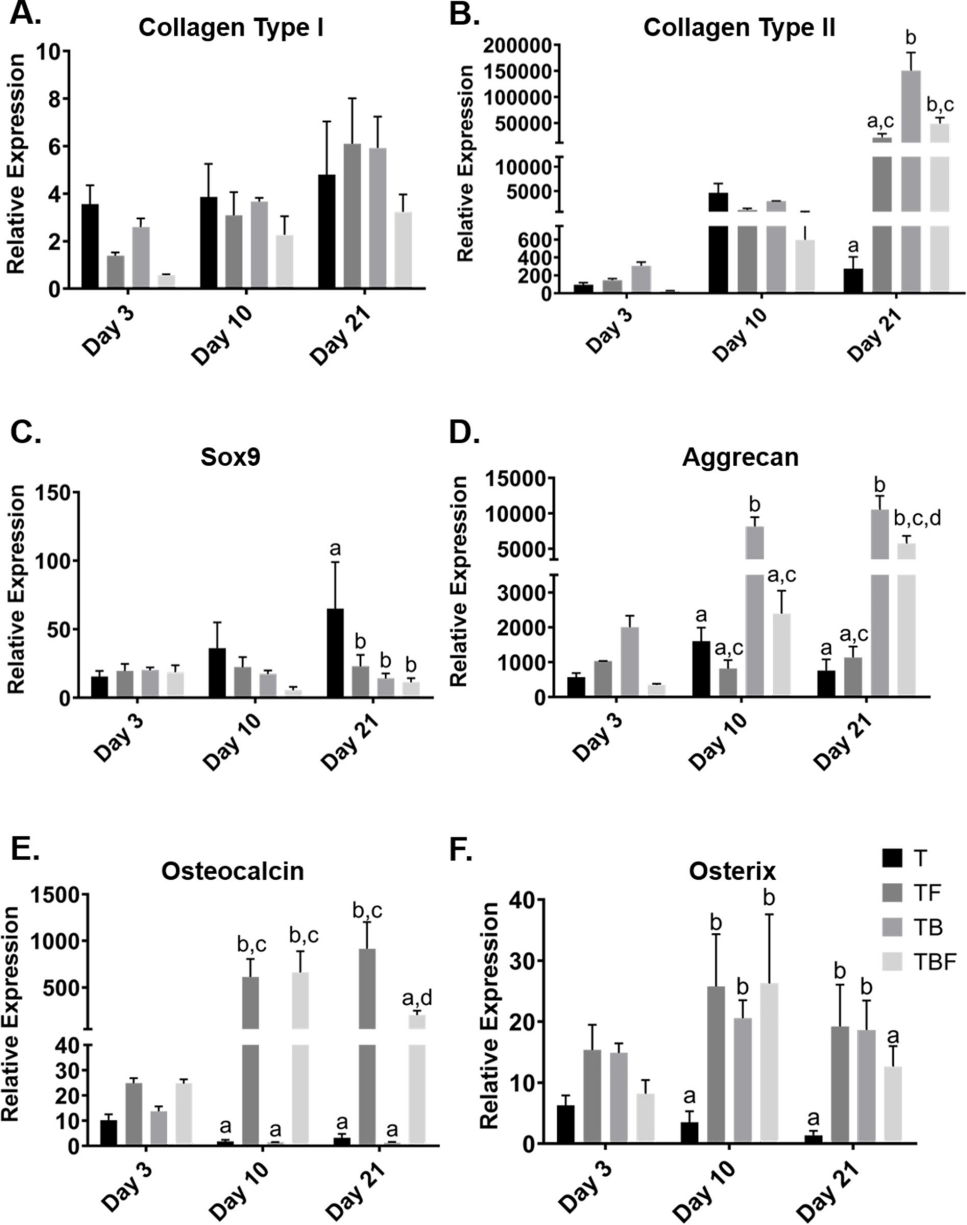
Transcriptional analysis of chondrogenic and osteogenic genes. Constructs were digested in collagenase to liberate cells from adjacent ECM. RNA was isolated for generation of cDNA. Quantitative PCR (qPCR) was performed on day 3, 10, and 21 constructs with relative expression normalized to RNA isolated from cMSCs on day 0 at the time of construct fabrication. A) There was a modest increase in *COL1A1 (Collagen Type I)* expression over time. B) *COL2A (Collagen Type II)* expression was significantly increased at day 21, with the greatest expression in cultures containing BMP-2 and/or bFGF. C) There was a modest increase in *SOX9* expression over time, with a significantly increased expression in treatment group T at day 21. D) There were significant differences in *ACAN (aggrecan)* expression at days 10 and 21, with treatment groups TB and TBF exhibiting the greatest relative expression. E) *BGLAP (Osteocalcin)* expression was significantly increased at days 10 and 21, with cultures treated with bFG exhibiting significantly greater expression (TF, TBF). F) There were significant increases in *SP7 (Osterix)* expression at days 10 and 21, with cultures treated with bFGF and/or BMP-2 (TF, TB, TBF) exhibiting higher relative expression of *SP7*.

Real-time (q)PCR was used to assess transcriptional changes in cMSC chondrogenic constructs using a panel of six genes of interest (Fig 7). Constructs were evaluated at day 3, day 10, and day 21 for transcriptional changes of *COL2A (Collagen Type II alpha chain)*, *ACAN (Aggrecan)* and the chondrogenic transcription factor *SOX9*. Constructs were assessed for transcriptional changes of *COL1A1 (Collagen Type I alpha chain)*, the extracellular matrix protein *BGLAP (Osteocalcin)*, and the osteogenic transcription factor *SP7 (Osterix)* [20].

There was a modest yet significant increase in the relative expression of *COL1A1 (Collagen Type I)* over time, regardless of treatment condition (p = 0.0001) (Fig 7A). Significant differences in *COL1A1* expression between treatment groups within each time point were not detected (p = 0.2559). In contrast, there was a dramatic increase in *COL2A* (Collagen Type II) expression over time, increasing approximately 50,000–100,000-fold over baseline at day 21 (p<0.0001). This was particularly noteworthy for treatment groups TF, TB, and TBF (Fig 7B). While there were no significant differences between treatment groups at day 3 or day 10, at day 21 treatment group T exhibited a reduced *COL2A* expression as compared to TB (p<0.0001) and TBF (p = 0.0036). *COL2A* expression was the highest in group TB, as compared to TF (p<0.0001), TBF (p<0.0001) or DT (p<0.0001). These results demonstrate that the system used in the present study markedly enhanced *COL2A* expression over time, with treatment group TB exhibiting the highest *COL2A* expression and group T exhibiting the lowest expression of *COL2A*.

Similar to the *COL2A* results, there was a substantial increase in the relative expression of *ACAN* over time (p = 0.0001), with expression increasing 5,000–10,000 for treatment groups TB and TBF (Fig 7B). While there were no significant differences in relative expression of *ACAN* at day 3, at day 10 *ACAN* expression in treatment group TB was significantly greater than group T (p<0.0001), TF (p<0.0001), or TBF (p<0.0001). A similar pattern was present at day 21. Treatment group TB had significantly greater relative expression of *ACAN* when compared to T (p<0.0001), TF (p<0.0001), or TBF (p = 0.0003). Moreover, group TBF had greater *ACAN* expression when compared to T (p = 0.0002) and TF (p = 0.0004). These results closely align with the *COL2A* expression (Fig 7B) and suggest that treatment groups TB and TBF resulted in the greatest degree of chondrogenic matrix gene expression.

The transcription factor *SOX9* exhibited a modest increase over time; however, differences across time points were not significant (p = 0.0644) (Fig 7C). There were no significant differences in *SOX9* expression at day 3 or day 10. At day 21, *SOX9* expression was significantly increased in treatment group T as compared to TB (p = 0.0053), TF (p = 0.0195), or TBF (p = 0.0036). When assessing transcriptional activity associated with osteogenic differentiation, there was a significant increase in the expression of *BGLAP (Osteocalcin)* over time (p = 0.0004) (Fig 7E). While we were unable to detect differences in expression of *BGLAP* at day 3, there was a significant increase in *BGLAP* expression at day 10 in group TBF as compared to T (p = 0.0007) and TB (p = 0.0007). Group TF had significantly higher *BGLAP* expression as compared to T (p = 0.0013) and TB (p = 0.0013). At day 21, there was a significant increase in *BGLAP* expression in group TF as compared to T (p<0.0001), TB (p<0.0001) and TBF (p = 0.0003). These results demonstrate that the treatment groups containing bFGF (TF and TBF) exhibited a significant increase in *BGLAP* expression over time, whereas groups lacking bFGF (T, TB) exhibited markedly reduced *BGLAP* expression.

Lastly, there was a significant increase in *SP7 (Osterix)* expression over time (p = 0.0014) (Fig 7F). While there were no significant differences in the relative expression of *SP7* at day 3, at day 10, the treatment group lacking BMP-2 and bFGF (T) had significantly lower *SP7* expression as compared to TB (p = 0.0280), TF (p = 0.0049) and TBF (p = 0.0041). At day 21, treatment group T had significantly lower *SP7* gene expression as compared to TB (p = 0.0265) and TF (p = 0.0218). While there was higher *SP7* expression in cultures treated with TBF as compared to group T, these differences were not significantly different (p = 0.1838). These results indicate that while BMP-2 and bFGF increased the expression of chondrogenic genes *COL2A* and *ACAN*, these growth factors also increased osteogenic transcriptional activity.

#### Histology

**Fig 8:**
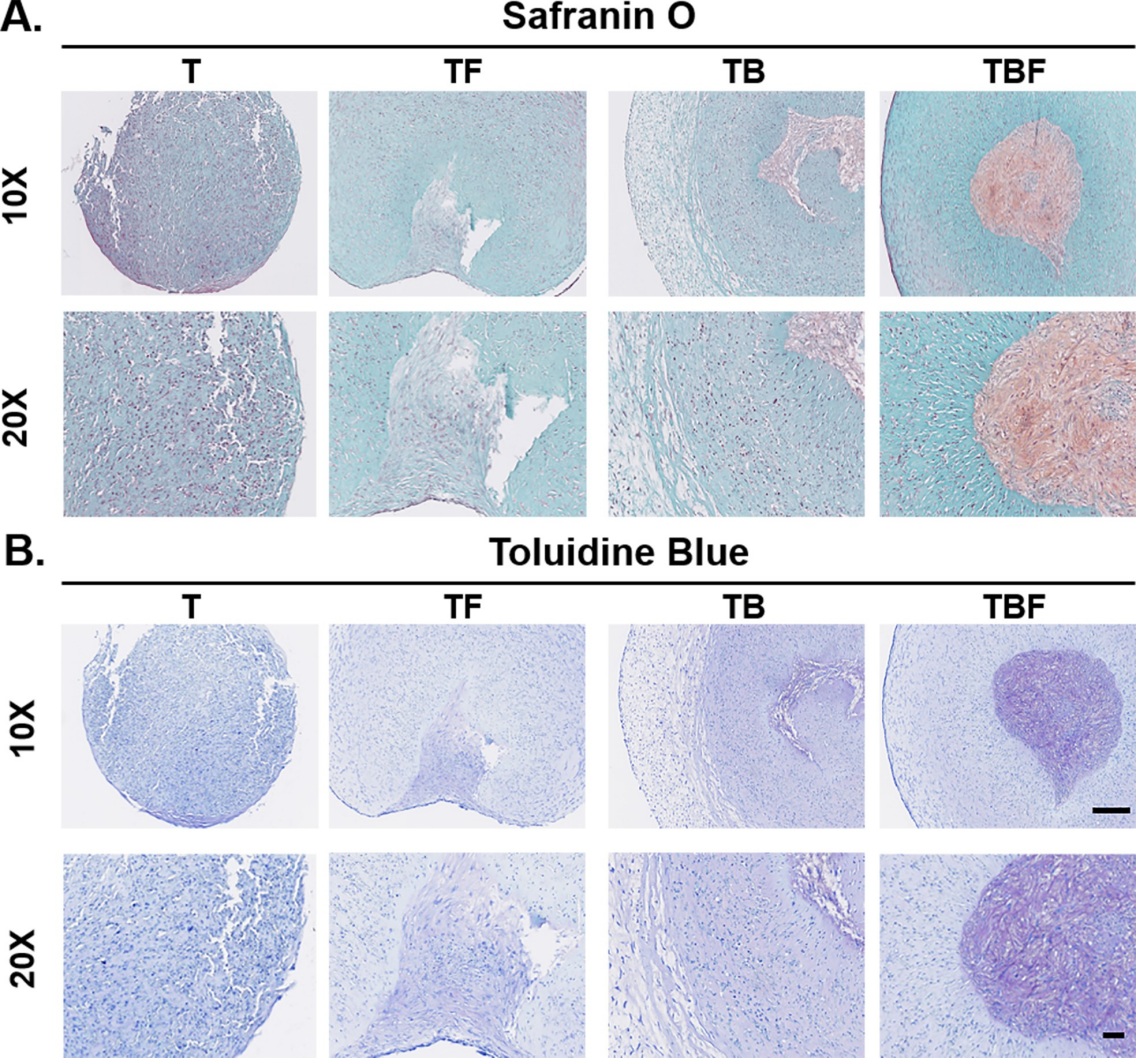
Safranin O and toluidine blue histology. Constructs were fixed and processed after 21 days of culture for histologic assessment with safranin O (A) or toluidine blue (B). Constructs treated with TGF-β3 (T) did not exhibit any evidence of chondrogenic differentiation. Constructs treated with TGF-β3 and bFGF (TF) exhibited small zones of faint proteoglycan deposition. Constructs treated with TGF-β3 and BMP-2 (TB) demonstrated focal regions of more convincing proteoglycan deposition, but contained a large rim of myxomatous tissue that lacked any proteoglycan staining. Constructs treated with TFG-β3, BMP-2, and bFGF (TBF) contained large zones of proteoglycan-rich ECM. Cells within these regions were spherical and produced lacunae-like structures and were morphologically similar to chondrocytes. Bars = 250 μm (10X) and 100 μm (20X).

Representative histologic images for the four treatment groups selected for in-depth analysis are provided in Fig 8. The histological appearance of the constructs at 21 days of culture was diverse. Treatment group T, which served as a control condition for the in-depth analysis did not exhibit any evidence of chondrogenic differentiation. At low magnification, constructs were spherical with cells evenly distributed throughout the constructs. These constructs lacked proteoglycan deposition. At higher magnification, cells adopted both spindle-shaped and rounded morphologies, but did not adopt a chondrocyte-like phenotype. Treatment group TF appeared histologically similar to T, although the constructs were larger in size, consistent with Fig 4A (group 2 and 4). In focal regions, there was weak proteoglycan staining, however cells did not develop lacunae or appear morphologically similar to chondrocytes. Treatment groups TB and TBF demonstrated the greatest degree of chondrogenic differentiation. The periphery of treatment group TB was somewhat unique to the other groups, containing a peripheral rim of poorly organized myxomatous tissue with a distribution of mesenchymal cells that did not stain for proteoglycans (Fig 8A and 8B, TB, 10X objective). There were occasional groups of mesenchymal cells organized as though they were differentiating down a chondrocyte lineage. In these areas, the extracellular matrix (ECM) stained blue to purple with toluidine blue and pink to orange with safranin O, confirming the presence of proteoglycans. Treatment group TBF exhibited a thin, spindle-shaped, darkly staining fibrous peripheral rim of tissue (Fig 8A and 8B, TBF, 10 X objective). TBF constructs exhibited large zones of abundant proteoglycan deposition. Cells within TBF constructs more consistently adopted a spherical morphology with more numerous lacunal-like structures within the proteoglycan-rich zones. These results indicate that constructs treated with chondrogenic media TB and TBF produced definitive regions of chondrogenesis and support the quantitative GAG results (Fig 6C, day 21).

#### Immunofluorescence

**Fig 9:**
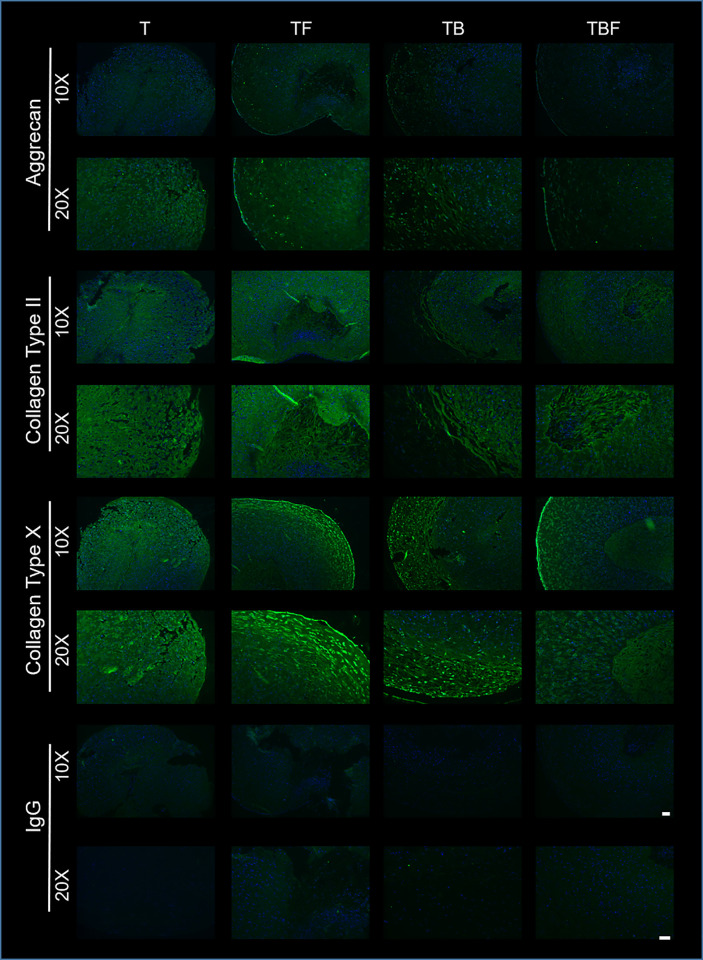
Immunofluorescence of select extracellular matrix (ECM) components. Matched sections from traditional histology (Fig 8) were assessed via immunofluorescence for aggrecan, Collagen Type II, and Collagen Type X. Primary antibodies were detected via a goat-anti-rabbit Alexafluor 488 secondary and nuclear staining was accomplished using DAPI. Negative control sections (IgG) were incubated with normal rabbit IgG prior to staining with Alexafluor 488-labelled secondary and DAPI. Aggrecan, Collagen Type II, and Collagen Type X were detected in all treatment groups. Aggrecan was detected more diffusely in groups T and TF, whereas in groups TB and TBF aggrecan signal was decreased throughout the ECM and localized to individual cells. Collagen Type II was detected within the ECM of all groups and was present both within the ECM and individual cells. Collagen Type X signal was greatest in groups TF and TB, whereas in TBF Collagen X signal was greatest in the peripheral rim of the construct. Bars = 100 μm (10X) and 50 μm (20X).

To further assess the ECM components of the constructs, immunofluorescence was performed for aggrecan, Collagen Type II, and Collagen Type X (Fig 9). Aggrecan was detected in all treatment groups. Interestingly, aggrecan signal appeared more diffusely distributed in treatment groups T and TF, whereas aggrecan staining was detected in the ECM but was also localized within the cells in treatment group TB and TBF. Collagen Type II was present in all treatment groups, with robust staining throughout constructs and enhanced staining in the regions of previously documented proteoglycan deposition (Fig 8). Collagen Type X was detected in all treatment groups, with robust staining in the treatment groups T, TF, and TB. Treatment group TBF exhibited the faintest staining, although a strong signal was detected around the periphery of TBF constructs.

#### TUNEL staining

Lastly, TUNEL staining was used to assess constructs for regions of apoptosis after 21 days in chondrogenic culture. Apoptosis was detected within the central region of treatment group T, with a particularly strong signal present in the core of this construct. TUNEL staining was not detected in treatment groups TF, TB, or TBF. These findings suggest that as late as 21 days, apoptosis was present within constructs treated with basal chondrogenic medium containing TGF-β3, whereas the presence of bFGF or BMP-2 prevented apoptosis.

## Discussion

The objectives of this study were to develop a 3D, serum-free system to facilitate canine chondrogenesis and to assess various concentrations and combinations of three known chondrogenic growth factors. Collagen Type I was selected as a biologic scaffold due to its availability, biocompatibility, spontaneous self-assembly [41], and use in prior chondrogenic studies [1,3]. As outlined in Fig 1, a system to generate 3D, serum-free, Collagen Type I constructs for cMSC chondrogenic assays was successfully developed. Given the challenges that have been described in the literature regarding cMSC chondrogenesis [19,42,43], this represents an important advance in the cMSCs field. The system was initially used to evaluate ten serum-free, chondrogenic induction media containing varying concentrations and combinations of TGF-β3, BMP-2, and bFGF (Figs 2A, 4 and 5). Results suggested that constructs treated with high concentrations of bFGF and BMP-2 were larger in size, exhibited a more classic spherical shape, and exhibited histologic evidence of chondrogenesis. Four of these chondrogenic media were selected for additional analyses.

**Fig 10:**
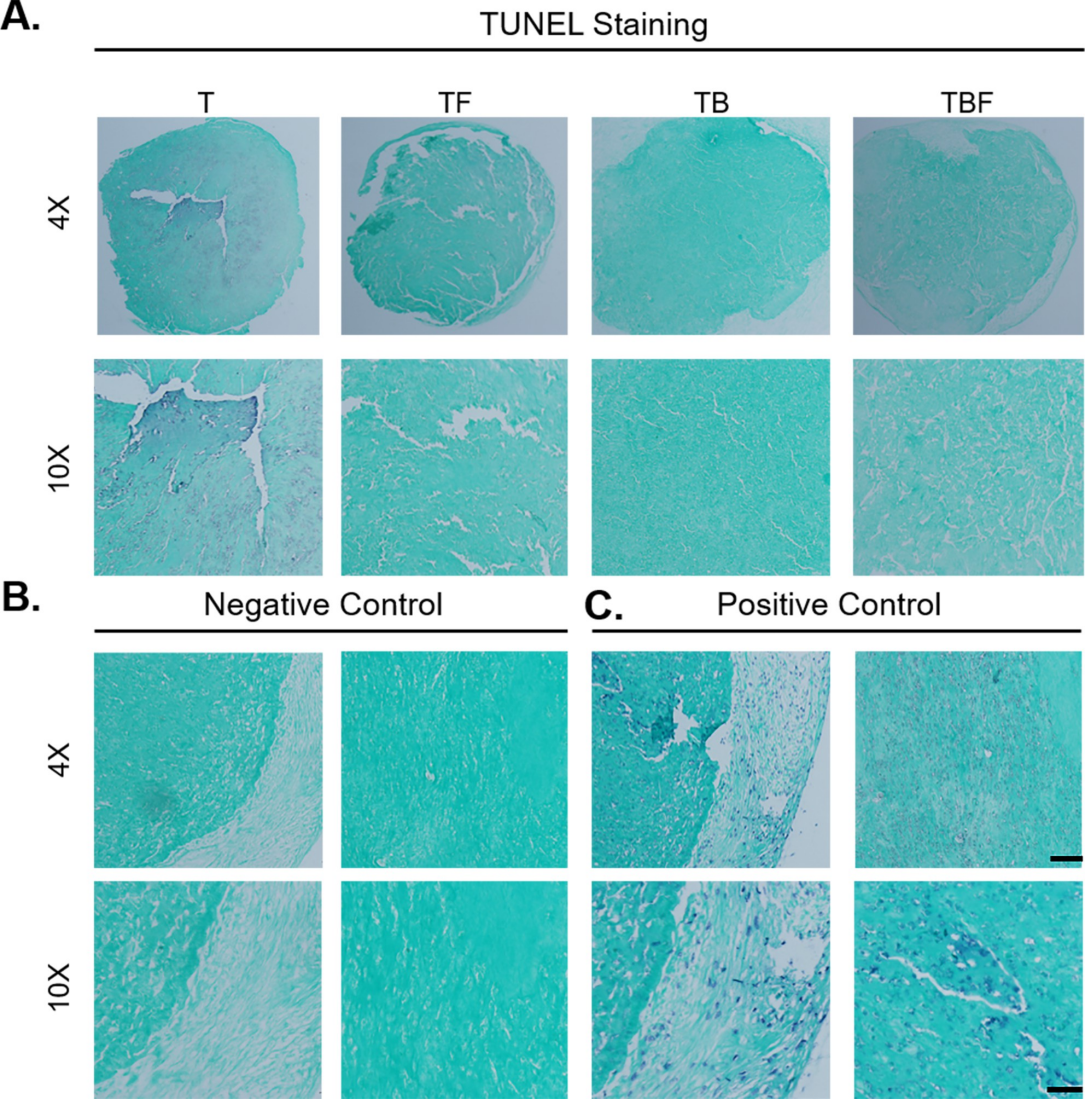
TUNEL staining. Constructs were cultured for 21 days prior to fixation and processing for histology and immunofluorescence as described in Figs 8 and 9. Sections for each treatment group were evaluated using the TUNEL method. All sections were counterstained with 0.5% Light Green stain. A) Positive staining was observed within the central region of treatment group T, with strong signal within the construct core. Staining was not observed in treatment groups TF, TB, or TBF. B) Negative controls were incubated with label solution lacking terminal transferase. C) Positive control slides were generated by applying recombinant DNase I to sections to induce DNA strand breaks, followed by incubation with TUNEL reaction mixture. Bars = 500 μm (4X) and 200 μm (10X).

Using live/dead staining and lactate dehydrogenase concentrations, constructs treated with media condition TBF (TGF-β3, BMP-2, bFGF) exhibited the lowest evidence of cytotoxicity (Fig 6A and 6B). Cultures treated with either TB and TBF also exhibited the highest GAG concentrations (Fig 6C). This was supported by qPCR results, which demonstrated a marked induction of the chondrogenic genes *COL2A* and *ACAN*, specifically in the treatment groups TB and TBF. Histologically, constructs treated with media condition TBF exhibited the greatest degree of chondrogenic differentiation (Fig 8). The presence of aggrecan, Collagen Type II, and Collagen Type X were confirmed with immunofluorescence (Fig 9). Lastly, there was no evidence of late-stage apoptosis when using TUNEL staining for any treatment group other than group (T) which was contained basal serum-free chondrogenic medium and TGF-β3 (Fig 10). Collectively, these results suggest that basal chondrogenic medium containing TGF-β3, BMP-2, and bFGF consistently generates superior chondrogenic constructs in this 3D, serum-free, Collagen Type I system.

Early in the culture period (day 3), constructs treated with media containing TGF-β3 underwent a dramatic condensation event (Fig 4A). This finding is similar to that observed *in vivo* as TGF-βs have been implicated in the modulation of N-cadherin expression and cell-cell interactions [44]. Tissue condensation has also been described in vascular biology during vascular regression, a phenomenon that occurs during completion of vascular morphogenesis and at the cessation of wound healing [45]. In that context, regression appears to be driven by matrix metalloproteinase (MMP) mediated degradation of the extracellular matrix [46]. N-cadherin and MMP expression and activity were beyond the scope of the present study, but may be of interest in future work.

Basic FGF (bFGF) is a key growth factor responsible for enhanced cell survival, proliferation, migration, and differentiation [9,22,33,47]. Basic FGF has been shown to enhance chondrogenesis of human MSCs [48,49]. Only a single study has evaluated bFGF during cMSC chondrogenesis [24]. Endo and colleagues isolated a specialized population of cMSCs, termed BM-PACs, and expanded these cells in the presence or absence of 10 ng/mL bFGF. A micromass technique was used to create small spheroids of 3 x 10^4^ cells that were cultured in chondrogenic medium supplemented with TGF-β1. BM-PACs expanded with bFGF exhibited increased transcription of *ACAN* and *COL2A*, increased Collagen Type II immunostaining, and stained strongly for proteoglycans with safranin O. The results of the present study support the findings of Endo, albeit a number of key differences exist between the two studies. In the present study, traditional bone marrow-derived cMSCs were evaluated. Cells were not exposed to bFGF during expansion, rather bFGF was provided during differentiation, where bFGF was included within the 3D Collagen Type I gels as well as culture media. Lastly, chondrogenic differentiation medium in the present study contained both TGF-β and BMP-2, which has been shown to enhance basal chondrogenesis. Chondrogenic media used by Endo and colleagues did not contain BMP-2 [24]. In the present study, bFGF reduced cytotoxicity in early cultures (Fig 6B) and when combined with TGF-β3 and BMP-2 resulted in the highest total GAG content (Fig 6C), greatest relative expression of *ACAN* and *COL2A* (Fig 7B and 7D), and produced the most convincing histologic evidence of chondrogenic differentiation (Fig 8, TBF). Inclusion of bFGF and/or BMP-2 was protective against late-stage apoptosis, as evidenced by TUNEL staining results. Thus, the findings of both Endo *et al*. and the present study suggest that bFGF enhances chondrogenesis of cMSCs. Future work will focus on the effect of expanding bone marrow-derived cMSCs in the presence or absence of bFGF prior to fabrication of chondrogenic constructs.

One important metric in development of articular cartilage tissue engineering constructs is GAG content and histologic evidence of GAG deposition. In the present study, constructs cultured in the presence of all three growth factors (TBF) contained significantly greater GAG concentration when compared to other groups. Quantitative GAG results were supported by immunofluorescence and suggest the increase in GAG was due to deposition within the ECM (Fig 8). It has previously been shown that inclusion of chondroitin sulfate and biotechnological chondroitin within cultures of chondrocytes resulted in maintenance of the chondrogenic phenotype and increased Collagen Type II expression [50,51]. Addition of GAGs to the 3D, serum-free, Collagen Type I system may further improve chondrogenic differentiation of cMSCs. This was beyond the scope of the present study.

This study was not without limitations. A single preparation of canine MSCs were used for all experiments. It would have been ideal to quantitatively assess cytotoxicity during live/dead staining (Fig 6A) with traditional fluorescent microscopy; however, this was not possible due to the high density of cMSCs within constructs (1 x 10^7^ cells/mL), the 3D nature of the constructs which produced tremendous background fluorescence, and the fact that disruption of the constructs during early phases of culture would have prevented late-stage histologic assessment. Although numerous growth factors have been linked to chondrogenesis, TGF-β, BMP-2, and bFGF were selected for evaluation in this study.

## Conclusion

It has been shown that the traditional micromass chondrogenic differentiation technique leads to inconsistent results with cMSCs. In the present study, we describe a 3D, serum-free, Collagen Type I system for facilitating cMSC chondrogenesis and generating constructs sized appropriately for clinical applications. Using this system, basal chondrogenic medium containing TGF-β3, BMP-2, and bFGF consistently generates superior chondrogenic constructs. These techniques will prove useful for investigators interested in studying the chondrogenesis of cMSCs. It may also prove useful as a foundation for translational studies in which the dog is used as model for treatment of focal cartilage defects with tissue engineering constructs.

## Supporting information

S1 Raw images (PDF)
