# Supplementary material for: A three-dimensional (3D), serum-free, Collagen Type I system for chondrogenesis of canine bone marrow-derived multipotent stromal cells (cMSCs)
Source: PLoS One. 2022 Jun 9;17(6):e0269571. doi: 10.1371/journal.pone.0269571 (PMC9182251; doi:10.1371/journal.pone.0269571)
Supplement: S1 Raw images — (PDF) [file pone.0269571.s001.pdf]

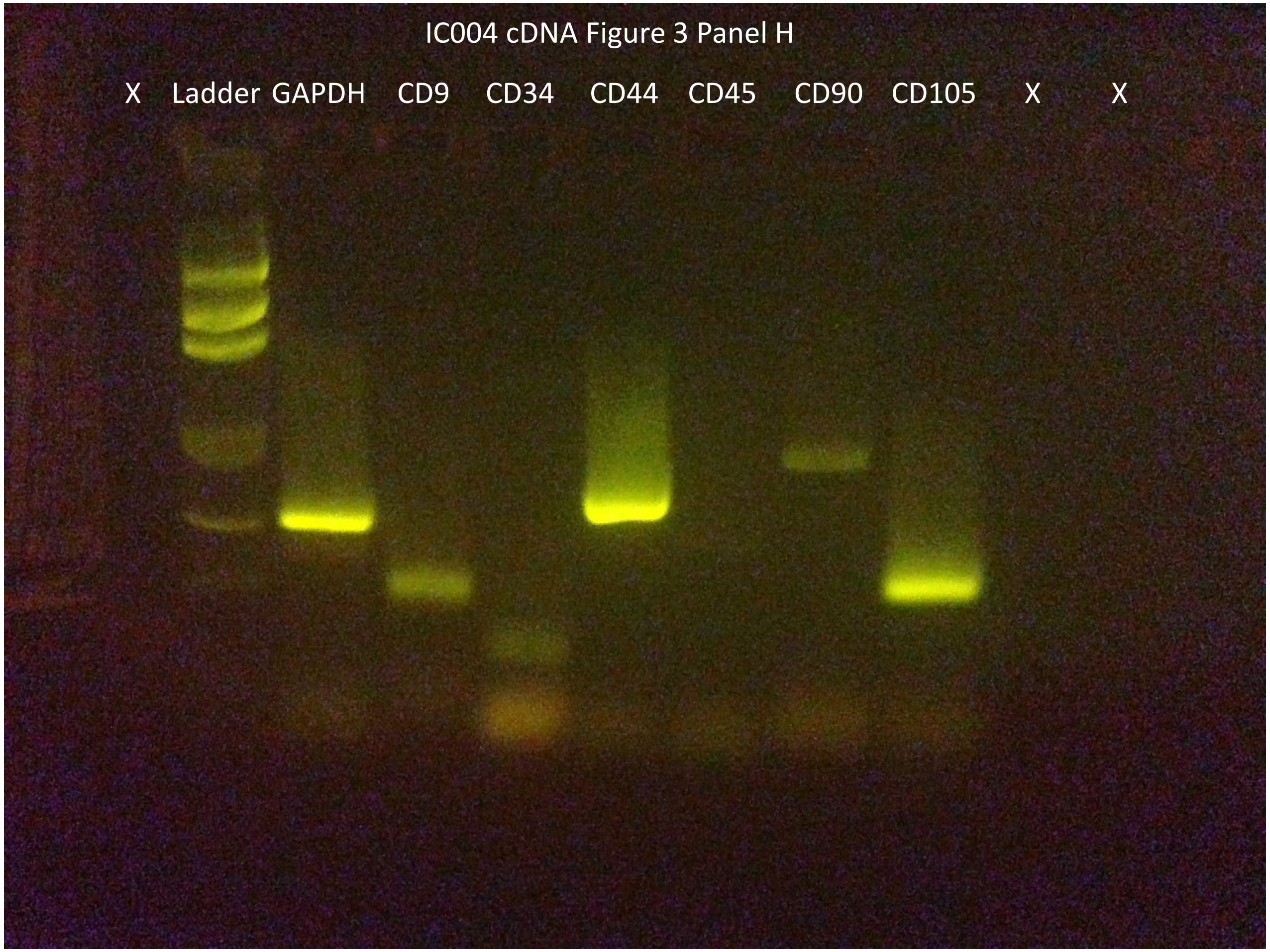
IC004 cDNA Figure 3 Panel H
X
Ladder
GAPDH
CD9
CD34
CD44
CD45
CD90
CD105
X
X

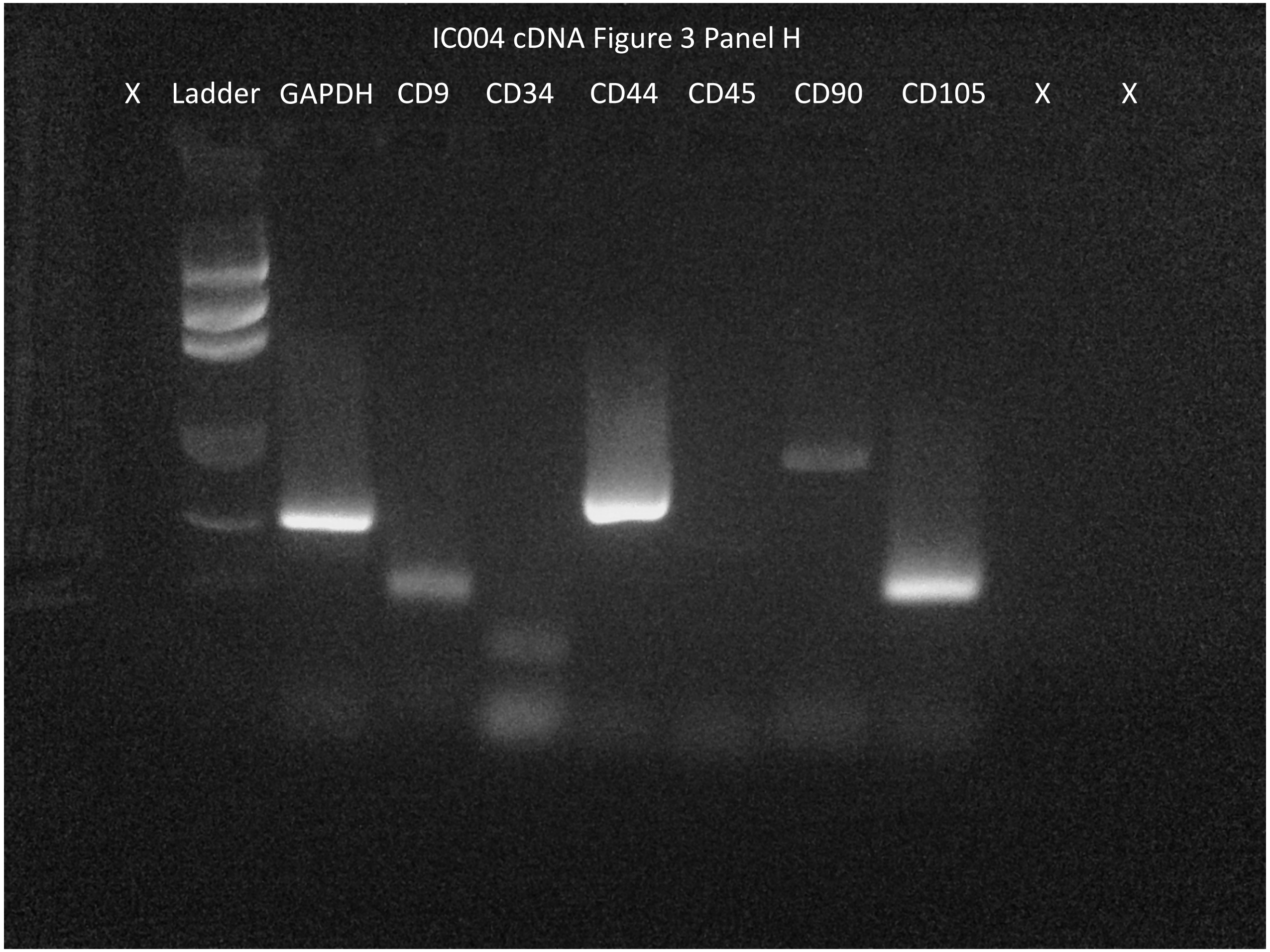
IC004 cDNA Figure 3 Panel H
X Ladder GAPDH CD9 CD34 CD44 CD45 CD90 CD105 X X
